# Vaccination against Influenza with Recombinant Hemagglutinin Expressed by *Schizochytrium* sp. Confers Protective Immunity

**DOI:** 10.1371/journal.pone.0061790

**Published:** 2013-04-23

**Authors:** Anne-Cécile V. Bayne, David Boltz, Carole Owen, Yelena Betz, Goncalo Maia, Parastoo Azadi, Stephanie Archer-Hartmann, Ross Zirkle, J. Casey Lippmeier

**Affiliations:** 1 Nutritional Lipids, DSM Nutritional Products, Columbia, Maryland, United States of America; 2 Division of Microbiology & Molecular Biology, IIT Research Institute, Illinois Institute of Technology, Chicago, Illinois, United States of America; 3 Complex Carbohydrate Research Center, University of Georgia, Athens, Georgia, United States of America; St. Jude Children's Research Hospital, United States of America

## Abstract

For the rapid production of influenza vaccine antigens in unlimited quantities, a transition from conventional egg-based production to cell-based and recombinant systems is required. The need for higher-yield, lower-cost, and faster production processes is critical to provide adequate supplies of influenza vaccine to counter global pandemic threats. In this study, recombinant hemagglutinin proteins of influenza virus were expressed in the microalga *Schizochytrium* sp., an established, fermentable organism grown in large scale for the manufacture of polyunsaturated fatty acids for animal and human health applications. *Schizochytrium* was capable of exporting the full-length membrane-bound proteins in a secreted form suitable for vaccine formulation. One recombinant hemagglutinin (rHA) protein derived from A/Puerto Rico/8/34 (H1N1) influenza virus was evaluated as a vaccine in a murine challenge model. Protective immunity from lethal challenge with homologous virus was elicited by a single dose of 1.7, 5 or 15 µg rHA with or without adjuvant at survival rates between 80–100%. Full protection (100%) was established at all dose levels with or without adjuvant when mice were given a second vaccination. These data demonstrate the potential of *Schizochytrium* sp. as a platform for the production of recombinant antigens useful for vaccination against influenza.

## Introduction

Influenza is an infectious disease caused by a few ever evolving quasi-species of the family *Orthomyxoviridae* against which human vaccination was first reported in 1937 [1]. Traditionally, influenza vaccines are created from inactivated or attenuated preparations of live virus cultured in chicken eggs. This approach suffers from several drawbacks. Most notably, it is a labor intensive process requiring 1 or 2 eggs per vaccine dose and no less than 6 months to scale-up for commercial manufacturing [2]. For any given influenza strain included in the annual trivalent vaccine, virus production usually requires artificial re-assortant strains and adaption regimens for growth in eggs. However, these manipulations contribute to the six-month timeline of influenza vaccine production and often result in vaccine antigens which no longer represent a perfect match to those of the parent strain, resulting in a mismatched vaccine. It is recognized that a transition from egg-based production systems to flexible cell-based and recombinant systems is desirable to continue long-term expansion of influenza vaccination programs and to better respond to sudden pandemics. To address these concerns, several groups have produced vaccines using cell-based systems, either by infection of cultured cells with live virus or by expression of influenza proteins from recombinant hosts including; vertebrate-derived cell lines [3], insect cell lines [4], yeast [5], filamentous fungi [6], higher plants [7], and bacteria [8]. Recombinant subunit vaccines are of particular interest as they can be used to eliminate many of the complications associated with currently available influenza vaccines (inactivated, split, and live-attenuated virus vaccines), having the potential to reduce scale-up periods to 12 weeks or less; half the time of egg-based systems [2]. Of the two influenza envelope glycoproteins, neuraminidase (NA) and hemagglutinin (HA), the latter elicits the highest proportion of virus-neutralizing antibodies which correlate to protection [9], [10]. Thus, HA has been the most popular target for recombinant expression using these alternative host cells, expression system platforms, and antigen delivery scaffolds [4]. Of the several influenza subunit vaccines being investigated, the most developed is manufactured using baculovirus-transfected insect cells, and has successfully completed Phase III clinical trials for consequent USFDA approval [11]. However, the acceptance of subunit vaccines composed of influenza envelope proteins has been confounded by limitations in antigen expression and presentation, glycosylation, and immune responses. To explore alternative approaches for the production of functional influenza antigens, this report investigates the expression and secretion of rHA using a novel, well-defined, commercially feasible, microalgal-based expression system.

Influenza HA binding to terminal sialic acids of host glycoproteins is required for viral entry into the host cell. HA is a type I fusogenic, membrane glycoprotein with an N-terminal signal sequence, a hydrophobic transmembrane anchor domain near its C-terminus, and a short cytoplasmic tail. The HA protein is synthesized as a precursor polypeptide (HA0), which folds and self-associates as non-covalently linked homotrimers in the endoplasmic reticulum, prior to transport through the Golgi apparatus to the plasma membrane. Each HA0 polypeptide is activated through cleavage by a host-encoded protease in the secretory pathway. The resulting polypeptides, HA1 and HA2, are linked by a single disulfide bond [12]. The HA protein is subject to other post-translational modifications before it is exported in association with the viral envelope. N-glycosylation sites in both the HA1 and HA2 vary in location and frequencies among individual strains of influenza, but some are conserved and play roles in immune evasion, virion export, receptor binding and protein folding [13]–[15]. HA is also acylated at a cysteine residue in the C-terminal tail which facilitates fusion pore formation of infectious virions [16]. In general, characteristics such as these influence the choice of transgenic host for expression of a vaccine antigen. Ideally, the required features of a target antigen will align with the known traits of a given host system. Preserving the critical, qualitative attributes of the HA protein may result in a more vigorous, multi-faceted immune response targeting multiple functional epitopes with enhanced neutralization potential.

*Schizochytrium* sp. is a robustly fermentable eukaryote, best known for its ability to accumulate large amounts of cellular triglyceride containing nutritionally important polyunsaturated fatty acids (PUFA); most notably, docosahexaenoic acid (DHA). The majority of *Schizochytrium*-derived triglyceride oil is used to fortify foods with DHA. *Schizochytrium* biomass has also found use in animal applications, most commonly as a blend in poultry or aquaculture feeds to increase levels of DHA in eggs or farmed seafood respectively. Ten tons per annum of triglyceride oil have been produced using *Schizochytrium*, after culture in volumes greater than 150,000 liters of fully defined media [17], [18]. The organism is highly efficient at consuming nutrients in the presence of low dissolved oxygen levels, with a reported peak rate of 9 doublings per day, which translates to more than 200 g/l of dry biomass accumulated in less than 4 days [19]. This productivity further translates to lower materials, energy and labor costs per unit mass produced in fermentors as compared to many other eukaryotic, cell based systems. A proprietary genome sequence and transformation system has been developed for *Schizochytrium* [20], which in prior studies has been used to create targeted gene disruptions via homologous recombination [21]. Several dominant, selectable markers have also been used for *Schizochytrium* transformation [20], [21] enabling a number of metabolic engineering projects centered on carbon metabolism and fatty acid biosynthesis [22]–[24]. *Schizochytrium* is not photosynthetic and is thus cultivated using fermentation methods. It is also characterized by bi-flagellate zoospores and by thin, non-retractable, filopodia-like extensions of the cytoplasm. These extensions complex to form net-like structures which are associated with an ectoplasmic net composed largely of exported polysaccharide [25], [26]. As a recombinant host expression system these *Schizochytrium* characteristics; cell wall flexibility and motility, may contribute to the export or extrusion of complex membrane bound molecules such as influenza HA.

In the present study, properties of recombinant HA derived from the classic strain, H1N1 influenza A/Puerto Rico/8/34 (PR8), were characterized, including glycosylation and proteolytic cleavage. Mice vaccinated with the PR8 rHA antigen were protected against lethal challenge with homologous virus. Additionally, we demonstrated expression of recombinant hemagglutinins derived from a number of influenza strains using *Schizochytrium*.

## Materials and Methods

### Strains, Vector Constructs, and Transformations

All vectors were constructed either in whole or in parts by Blue Heron Biotechnologies (Bothell, WA) or DNA 2.0 (Menlo Park, CA) and supplied as purified frozen DNA. All HA protein coding regions were codon optimized for expression in *Schizochytrium* sp. ATCC 20888 using the proprietary algorithms of either DNA 2.0 (Menlo Park, CA, USA) or Blue Heron Biotechnologies (Bothell, WA, USA). Accession numbers for each of the vector constructs harboring various HA genes are listed in Table 1. Vector elements including promoters, terminators and selectable markers have been described elsewhere [27]. Cultures of *Schizochytrium* sp. ATCC 20888 were transformed with supercoiled vector by particle bombardment as previously described [21] and as follows. *Schizochytrium* cultures were grown from cryostocks in 50 ml volumes of “M50–20” media consisting of 20 g/l glucose, 12.5 g/l NaCl, 2.5 g/l MgSO_4_·7 H2O, 0.4 g/l KH_2_PO_4_, 0.5 g/l KCl, 0.05 g/l CaCl_2_·2 H_2_O, 0.5% modified PB26 metals and 0.1% modified PB26 vitamins (v/v). Modified PB26 vitamins consisted of 0.5 µg/ml biotin, 100 µg/ml thiamine, and 500 µg/ml cyanocobalamin. Modified PB26 metals were adjusted to pH 8.0 and consisted of 6 g/l Na_2_-EDTA, 0.29 g/l FeSO_4_·7 H_2_O, 6.84 g/l H_3_BO_3_, 0.86 g/l MnCl_2_·4 H_2_O, 60 mg/l ZnCl_2_, 26 mg/ml CoCl_2_·6 H_2_O, 5 mg/l Na_2_MoO_4_·2 H_2_O, 2 mg/l CuSO_4_·5 H_2_O, and 52 mg/l NiSO_4_·6 H_2_O. PB26 stock solutions were filter sterilized separately and added to the broth after autoclaving. Glucose, KH_2_PO_4_, and CaCl_2_·2 H_2_O were each autoclaved separately from the broth ingredients. All media components and antibiotics were purchased from Sigma (St. Louis, MO). Cultures of *Schizochytrium* sp. were grown to log-scale before transformation with ∼5 µg of plasmid coated on M10 tungsten particles delivered via biolistic particle bombarder (BioRad, Hercules, CA, USA) using a 1100-psi rated burst disk and with the culture dish on the second shelf slot. Primary transformants were selected on solid M2B media [21] containing 15 g/l agar (VWR, West Chester, PA) and 50 µg/ml paromomycin. All primary transformants were manually transferred to grid-plates containing paromomycin before being used to inoculate a 50 ml culture of M50–20 for preparation of 40% glycerol cryostocks. Primary transformants were further verified by polymerase chain reactions directed to vector elements and performed on gDNA extracted as previously described [21].

**Table 1:** Hemagglutinin sequences and corresponding vectors containing the codon-optimized nucleic acid sequences with their accession numbers.

Hemagglutinin source	HA Accession #	Vector	Vector Accession #	HAU
A/Puerto Rico/8/1934 (H1N1)	AAM75158	pCL0143	KC218919	512
A/Puerto Rico/8/1934 (point mutant)	n/a	pCL0154	KC218921	512
A/New Caledonia/20/1999 (H1N1)	ABW80979	pCL0161	KC218923	512
A/Hong Kong/483/1997 (H5N1)	AAC32099	pCL0160	KC218922	16
B/Malaysia/2506/2004	ACO05957	pCL0153	KC218920	512

The highest recorded activities derived from a shake flask culture of a single transformant are listed as hemagglutination activity units (HAU) per 50 µl of cell free supernatant.

### Shake Flask and Fermentation Culture Conditions for HA Expression

Supernatants containing recombinant HA proteins were prepared from fermentors or baffled shake flask cultures. Shake flask cultures contained 50 ml of protein-free “SPFM” media, inoculated from a colony or M50–20 culture aliquot. SPFM media contained 13.62 g/l Na_2_SO_4_, 0.72 g/l K_2_SO_4_, 0.56 g/l KCl, 2.27 g/l MgSO_4_·7 H_2_O, 3 g/l (NH_4_)2 SO_4_, 0.19 g/l CaCl_2_·2 H_2_O, 3 g/l Na-glutamate, 21.4 ml/l 2-(N-morpholino)ethanesulfonic acid (MES) (100 mM, pH 6), 0.4 g/l KH_2_PO_4_, 50 g/l glucose, 2 ml/l of SSFM-PB26 trace metals, and 1 ml/l of SSFM-PB26 vitamins. SSFM-PB26 vitamin stock contained 0.16 g/l vitamin B12, 9.75 g/l thiamine, and 3.33 g/l Ca-pantothenate. SSFM-PB26 trace metals stock was set to pH 2.5 with HCl and contained 1 g/l citric acid, 5.15 g/l FeSO_4_·7 H_2_O, 1.55 g/l MnCl_2_·4 H_2_O, 0.965 g/l ZnSO_4_·7 H_2_O, 0.02 g/l CoCl_2_·6 H_2_O, 0.02 g/l Na_2_MoO_4_·2 H_2_O, 1.035 g/l CuSO_4_·5 H_2_O, and 1.035 g/l NiSO_4_·6 H_2_O. Trace metals, vitamins, glucose, KH_2_PO_4_, and CaCl_2_·2 H_2_O were all filter sterilized and added to the media after the remaining ingredients were autoclaved. Flask cultures were grown for 2–5 days at 27°C with constant, 200 rpm agitation before cell-free supernatants (CFS) were collected by centrifugation at 5000 *g*.

Supernatants containing PR8 HA obtained using 10 l fermentors were derived from cultures grown with modified SPFM media containing the same salts as above, with the exception of 6 g/l (NH_4_)2 SO_4_, 6 g/l Na-glutamate, and omitted MES. All vessels were autoclaved for 2 hours at 120°C and to these, 50 g/l glucose, 0.8 g/l KH_2_PO_4_, 0.19 g/l CaCl_2_·2 H_2_O, 4 mg/l citric acid, 20.6 mg/l FeSO_4_·7 H_2_O, 6.2 mg/l MnCl_2_·4 H_2_O, 3.9 mg/l ZnSO_4_·7 H_2_O, 0.08 mg/l CoCl_2_·6 H_2_O, 0.08 mg/l Na_2_ MoO_4_·2 H_2_O, 4.14 mg/l CuSO_4_·5 H_2_O, and 4.14 g/l NiSO_4_·6 H_2_O were added. These additions were autoclaved for 50 minutes at 120°C. Finally, a vitamin stock solution containing 0.32 g/l vitamin B12, 19.5 g/l thiamine, and 6.66 g/l Ca-pantothenate was filter sterilized and 10 ml of this stock was added to the media. The fermentation was provided with a constant airflow of 8 l/minute supplied throughout. Dissolved oxygen was set at 20%. Vessel agitation was allowed to adjust from a range of 357–900 rpm as needed to maintain the dissolved oxygen set point. Temperature was maintained at 22.5°C. The pH was set at 6.75 and maintained through control loop additions of either 4 N NaOH or 3 N H_2_SO_4_. Broth was collected at 36 hours and centrifuged at 5000 *g*.

### Hemagglutination Assay

HA assays were performed as described previously with modifications [28]. Briefly, 50 µl of doubling dilutions of CFS in PBS were prepared in a 96-well microtiter plate. Equal volume of an approximate 1% solution of chicken red blood cells (Fitzgerald Industries, Acton, MA) in PBS, pH 7.4, was then added to each well followed by incubation at room temperature for 30 minutes. The degree of agglutination was then analyzed visually. The hemagglutination activity unit (HAU) is defined as the highest dilution that causes visible hemagglutination in the well. A recombinant HA protein derived from Influenza A/Vietnam/1203/2004 (H5 N1) (Protein Sciences Corporation, Meriden, CT, USA) was used as positive control for HA activity.

### Protein Analysis and Immunoblotting

*Schizochytrium* cultures were transferred to 50 ml conical tubes and centrifuged at 3000 *g* or 4500 *g* for 15 min. The supernatants resulting from this centrifugation were in some cases further centrifuged at 100,000 *g* for 1 hour. CFS or other protein samples were separated by sodium dodecyl sulfate-polyacrylamide gel electrophoresis (SDS-PAGE) on a NuPAGE® Novex® 12% bis-tris gel (Invitrogen, Carlsbad, CA) under reducing conditions with 3-(N-morpholino) propanesulfonic acid - sodium dodecylsulfate (MOPS-SDS) running buffer, unless indicated otherwise in the text. The proteins were then stained with SimplyBlue Safe Stain (Invitrogen, Carlsbad, CA, USA) or transferred onto polyvinylidene fluoride membrane and probed for the presence of HA protein with anti-PR8 virus antiserum from rabbit, catalog number 11684-RP01 (Sino Biological Inc., Beijing, China), followed by anti-rabbit IgG (Fc) secondary antibody coupled to alkaline phosphatase (Promega Corporation, Madison, WI). The membrane was then treated with 5-bromo-4-chloro-3-indoyl-phosphate/nitroblue tetrazolium solution according to the manufacturer's instructions (KPL, Gaithersburg, MD). Protein sequences were confirmed by mass spectrometry (Finnigan LTQ linear ion trap) according to the protocol developed by the Mass Spectrometry Laboratory for Protein Sequencing at the Lerner Research Institute (Cleveland Clinic, Ohio) [29] and by Edman degradation using a 494 Procise Protein Sequencer/140 C Analyzer (Applied Biosystems, Inc., Carlsbad, CA, USA) according to the protocol developed by Iowa State University Protein Facility [30].

### Glycosylation Analysis

A CFS fraction from a *Schizochytrium* culture containing PR8 HA was run on NuPAGE® Novex® 12% bis-tris gel (Invitrogen, Carlsbad, CA, USA) under non-reducing conditions with MOPS buffer and the resulting gel was stained with SimplyBlue Safe Stain. The HA protein was excised from the gel and alternately treated with destain solution (DS) comprised of 40 mM ammonium bicarbonate, and 100% acetonitrile, until the color turned clear. Destained gel was re-swelled in 10 mM dithiothreitol (DTT) in 40 mM ammonium bicarbonate at 55°C for 1 hour. The DTT solution was exchanged with 55 mM iodoacetamide and incubated in the dark for 45 minutes. Incubation was followed by two washes with DS before being dehydrated in acetonitrile and subjected to in-gel deglycosylation [31]. The dehydrated gel sections were hydrated at 4°C with 50 mM sodium phosphate, pH 7.5 containing PNGase F (New England Biolabs, Ipswich, MA, USA) and then incubated at 37°C overnight to release N-glycans. Free glycans were extracted from the gel in series with 20% acetonitrile in 5% formic acid, 50% acetonitrile in 5% formic acid, and finally 80% acetonitrile in 5% formic acid. The sample solutions were dried, combined for passage through a C18 sep-pak cartridge (Resprep, Bellefonte, PA, USA), eluted in 5% acetic acid to remove contaminants, and dried again by lyophilization. Released N-linked oligosaccharides were permethylated [32] and analyzed by nanospray ionization mass spectrometry (NSI-MS^n^) using a LTQ Orbitrap XL mass spectrometer (Thermo Fisher, Waltham, MA, USA). Specifically, permethylated glycans were dissolved in 1 mM NaOH in 50% methanol and infused directly into the instrument at a constant 0.5 µl/min flow rate. A full Fourier transform MS spectrum was collected at a resolution of 30,000. The capillary temperature was set at 210°C and MS analysis was performed in the positive ion mode. For total ion mapping (automated MS/MS analysis), m/z range 800 to 2000 was scanned using ion trap MS mode in successive 2.8 mass unit windows that overlapped the preceding window by 2 mass units.

### Protein Purification

Cells of *Schizochytrium* expressing HA were cultivated under the fermentation conditions described above and centrifuged for 15 min at 3000 *g.* The CFS was then diluted 1∶1 in a solution containing 20 mM sodium phosphate pH 7.0, 5% glycerol, 1 mM EDTA, and 0.2% Triton™ X-100. The mixture was filtered (0.45 µm pore size) and transferred to an anion-exchange spin column (Thermo Scientific Pierce Biotech, Fisher Scientific, Rockford, IL USA) previously equilibrated with 20 mM sodium phosphate pH 7.0, 5% glycerol, and 0.1% Triton™ X-100. The loaded sample was centrifuged according to the manufacturer’s instructions. The resulting flow-through was concentrated and buffer was exchanged with phosphate-buffered saline (PBS), pH 7.4 by ultrafiltration using Amicon centrifugal devices (Millipore, Billerica, MA).

### Immunizations and Animal Model

Female BALB/c mice (6–8 weeks old) were purchased from Charles River Laboratories (Wilmington, MA, USA). The purified HA antigen was stored at ≤ −65°C before being formulated for injection. Vaccine and vehicle control formulations were prepared immediately prior to injection such that each dose (15, 5, 1.7 and 0 µg of HA) could be administered i.m. in 100 µl volumes bilaterally (50 µl/quadricep). Each dose was diluted in PBS to the appropriate concentration, or for corresponding adjuvant cohorts, to a concentration 2 fold higher for emulsification with Addavax™ (Invivogen, San Diego, CA, USA) at a 1∶1 ratio (v:v) according to the manufacturer’s instructions. Placebo (PBS) and adjuvant were also mixed at a ratio of 1∶1. The study design and experimental groups are detailed in the results section. Precisely, groups 4 and 11 were immunized with the rHA-Addavax mixture. Groups 1–3 were given single dose immunizations of descending mass. Groups 7–9 were given identical initial doses which were then followed by a second dose 3 weeks later. Group 11 was established to test mice subjected to all of the above variables combined. Groups 5, 6, and 10 were given volume-matched placebo injections of a single PBS dose, a single PBS-Addavax dose, or two doses of PBS respectively.

All mice were lethally challenged 21 days after the final vaccination. Mice were anesthetized i.p. with ketamine (100 mg/kg)/xylazine (10 mg/kg) and intranasally inoculated with 3000 TCID_50_ (5 MLD_50_) of infectious PR8 virus. The mice were then weighed and monitored daily for signs of disease. Mice were humanely euthanized with CO_2_ if their body weight dropped to 75% of baseline weights. All studies were conducted under applicable laws and guidelines and were approved by the IIT Research Institute Animal Care and Use Committee.

### Serological Assays

Serum samples were treated with receptor destroying enzyme (RDE) (Denka Seiken, Tokyo, Japan) overnight at 37°C and diluted 1∶10 with phosphate buffered saline (PBS). RDE treated samples were serially diluted into eight two-fold dilutions (1∶10 to 1∶20,480) in PBS and tested in a hemagglutination inhibition (HI) assay with 0.5% chicken red blood cells. The reciprocal of the highest dilution of serum or plasma that completely inhibited hemagglutination was determined to be the HI titer. For computational purposes, titers of <1∶10 were assigned a value of 1∶5. Titer values were log_2_ transformed prior to conducting statistical analysis and expressed as geometric mean titers (GMT).

### Bronchoalveolar Lavage Virus Titers

On days 3 and 7 after inoculation with PR8 influenza virus, 1–3 mice in each experimental and control group were sacrificed. Bronchoalveolar lavage (BAL) fluids were collected in two 1 ml washes with PBS. BAL samples were stored at ≤ −65°C until live virus titer determination by TCID_50_ assay in Madin-Darby Canine Kidney (MDCK) cells. MDCK cells were inoculated with serial dilutions of BAL fluid and incubated at 37°C for 48 hours. A hemagglutination assay was performed and virus titers (log_10_ TCID_50_/ml) were calculated by the Reed and Muench method [33].

### Statistical Analysis

Comparison of survival between groups of mice was performed with the log rank test on the Kaplan–Meier survival data. Comparison of the antibody and viral titer data was performed using analysis of variance for multiple comparisons with Systat (Systat, Chicago, IL). Error bars represent standard deviation, and statistical significance was defined as P<0.05.

## Results

### Expression of Viral Glycoproteins by *Schizochytrium* sp

To determine if *Schizochytrium* is capable of expressing intact viral glycoproteins, the full-length reading frames of two H1, one H5 and one influenza B hemagglutinins were codon optimized and incorporated within a *Schizochytrium* expression vector in a context that permitted expression under control of the *Schizochytrium* EF-1 promoter and PFA3 terminator. The strains from which the HA protein sequences were derived as well as the accession numbers of the vector constructs used to express them, are listed in Table 1. The constructs were specifically designed to bias for ectopic integration events. No fewer than ten transgenic clones resulting from transformation with each vector were cultured for two days under standard conditions for *Schizochytrium* and cell free supernatant (CFS) preparations were screened by hemagglutination assay for recombinant protein activity. Results indicated that the proteins were expressed and exported to the extracellular space in an active form. Among all transformants, hemagglutination activities ranged from 16 to 512 HAU/50 µl of CFS (Table 1).

### Production of HA from Influenza PR8 by *Schizochytrium* sp

**Figure 1:**
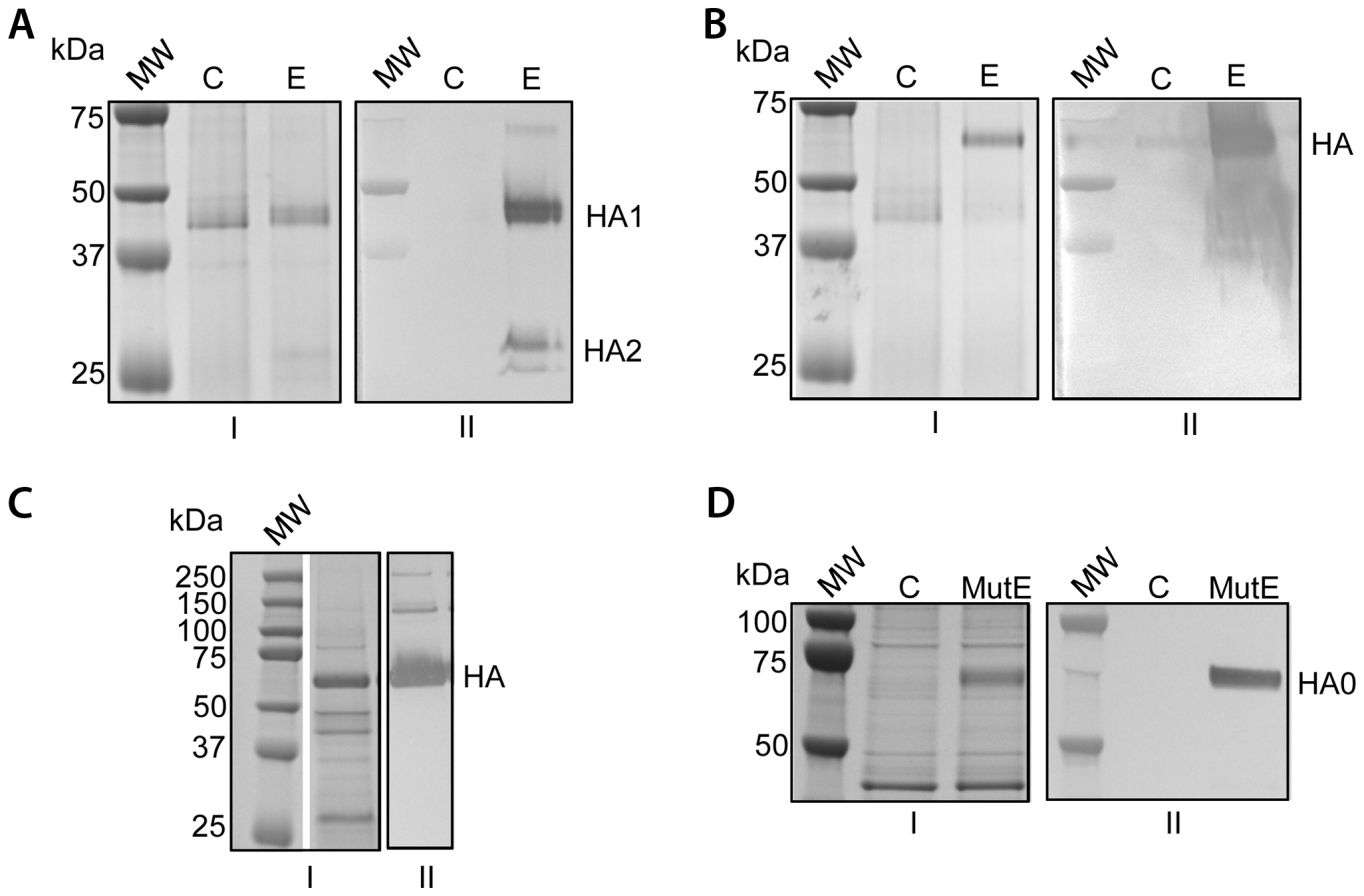
Production of hemagglutinin from Influenza strain A/Puerto Rico/8/34 by *Schizochytrium* sp. SDS-PAGE analysis of cell-free supernatants under reducing (A and D) and non-reducing (B and C) conditions and corresponding immunoblots with anti-Influenza A virus antibody are shown. Panels “I” are gels stained with Coomassie blue and panels “II” are the corresponding immunoblots. (A) Analysis of cell-free supernatants from *Schizochytrium* control “C” and experimental “E” strains showing the presence of HA subunits 1 and 2 under reducing conditions HA1 and HA2). (B) Analysis of cell-free supernatants from *Schizochytrium* control “C” and experimental “E” strains showing the presence of HA under non-reducing conditions. (C) SDS-PAGE analysis (non-reducing) of the *Schizochytrium* HA fraction used for the mouse challenge study. (D) Analysis of cell-free supernatants from *Schizochytrium* control “C” and mutant experimental “MutE” strains showing the presence of an uncleaved form of HA (HA0) under reducing conditions.

Of the various hemagglutinins screened by HA assay, the one derived from PR8 virus (H1 serotype) was selected for further characterization. Transformants of the vector pCL0143 were screened as described above and by immunoblot analysis. A range of hemagglutination activities were observed among the pCL0143 transformants (0–512 HAU). The strain generating the highest activity was designated 143-9. CFS prepared from a culture of this clone was analyzed by immunoblot under reducing conditions and showed the presence of two reactive bands corresponding to the expected molecular weights of HA1 and HA2 (Fig. 1A, lane “E”). The identity of the bands was confirmed by peptide sequence analysis (data not shown). Immunoblot analysis of the CFS under non-reducing conditions resulted in a single reactive band corresponding to the disulfide-bonded HA heterodimer (Fig. 1B). The migration of these bands agreed well with the predicted masses of HA1 (36.7 kDa), HA2 (25.1 kDa) and of the HA heterodimer (63.4 kDa). Centrifugation of the CFS from a shake flask culture at 100,000 *g* for 60 minutes indicated that the hemagglutinin is mostly associated with the insoluble fraction. The soluble fraction displayed 16 HAU whereas the insoluble fraction yielded 256 HAU. N-terminal sequence analysis also confirmed that the signal peptide is cleaved as would be expected for export via the secretory pathway (data not shown).

Clone 143-9 was also cultured in a 10 liter fermentor overnight in protein-free media according to the conditions described herein. Fermentation-derived CFS was assayed for HA activity which was notably improved (4096 HAU) as compared to CFS derived from shake flask cultures (512 HAU). PR8 HA antigen from fermentation culture supernatants of 143-9 were partially purified (approximately 20% of the total protein) for glycosylation analysis and for formulation as a vaccine as described in the Materials and Methods section. This material was also analyzed by non-reducing SDS-PAGE and by immunoblot (Fig. 1C).

To verify that the protein was cleaved at the appropriate location, a point mutant variant of the HA gene, that specifically changed the arginine residue of the conserved R∧G hemagglutinin cleavage site to a serine, was engineered and inserted in the same backbone vector as was used for pCL0143 construction. This new vector was named pCL0154 and was used to transform *Schizochytrium*. The resulting transgenic strains also exported HA to the extracellular medium, as indicated by activity assays performed on isolated CFS. Immunoblot analysis of one of those strains (“MutE”) indicated that the protein migrated as a single band in reducing conditions (Fig. 1D). This finding suggests that *Schizochytrium* sp. possesses an endogenous protease capable of mediating HA0 cleavage; a property shared with susceptible hosts of influenza viruses.

### Characterization of PR8 HA Glycosylation

**Figure 2:**
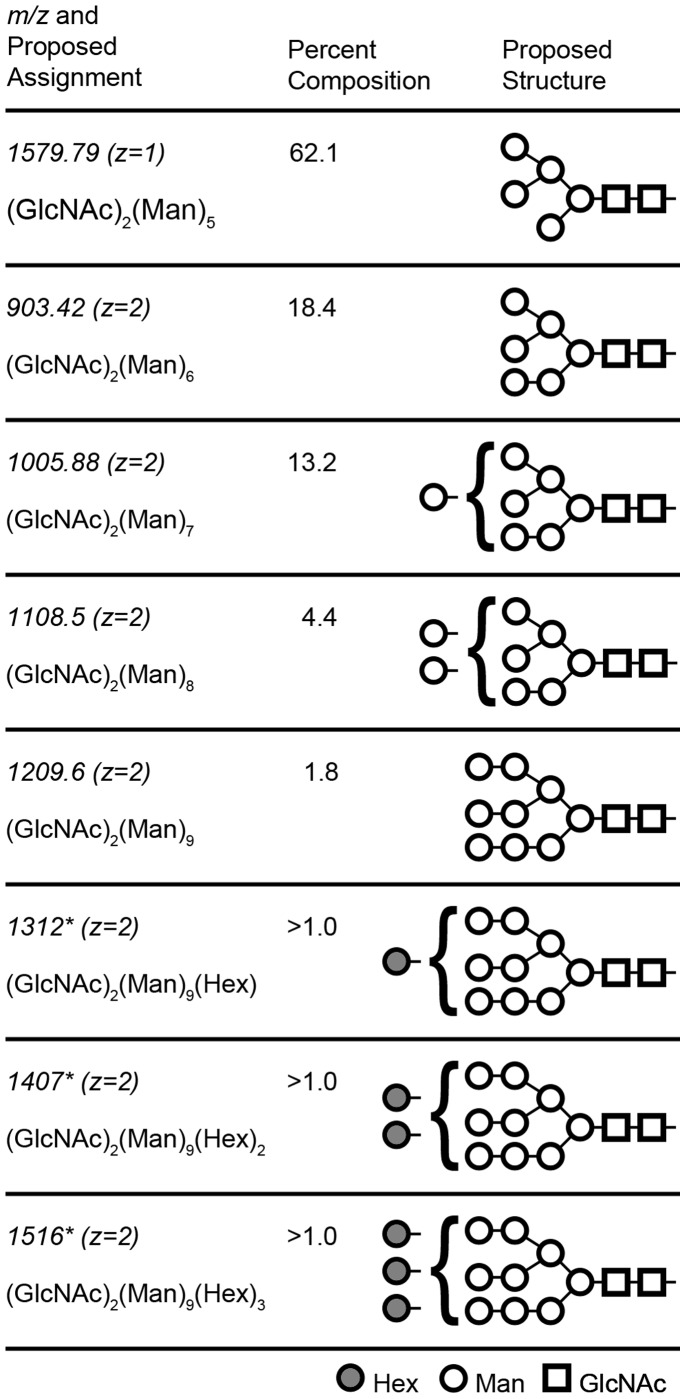
Permethyated glycans released from glycosylated rHA *Schizochytrium* cell-free supernatant. Given weights include addition of sodium, and account for permethylation. *Observed during total ion mapping (TIM), m/z value refers to the 2 mass unit window in which the noted MS/MS loss was observed. Hexose (Hex), mannose (Man), and N-acetylglucosamine (GlcNAc) residues are represented by gray circles, open circles, and open squares, respectively.

Influenza hemagglutinin is a glycosylated protein which binds terminal sialic acid residues of host protein glycoforms. Mass spectrometry (NSI-MS^n^) of the released and permethylated N-glycans was used to determine the glycoform structures associated with purified rHA, and to determine how they might differ from glycoforms made by native hosts of influenza (Figure 2). A series of ions were detected which correspond to oligosaccharide Man_5–9_GlcNAc_2_ structures. The identities of glycan signals observed in the full MS spectrum were confirmed by subjecting the signals to fragmentation in MS/MS analysis. Typical high mass fragmentation patterns were detected for each of the glycans, with predominant fragmentation at the bisecting GlcNAc residues. A trace amount (less than 1%) of hyper-mannosylation was also detected at 1312, 1407 and 1516 (Z = 2) corresponding to (GlcNAc)_2_(Mannose)_9_(Hex)_1–3_, respectively. The precise identity of the terminal hexose found among these structures could not be inferred by these analyses because permethylated hexoses all share the same mass (MW 204). No fucose or xylose residues were detected among any glycoforms. Sialic acid residues were also not detected in this analysis and a survey of a proprietary, *Schizochytrium* genome database found no genes related to sialic acid biosynthesis.

### Immunization and Infectious Challenge of Mice

**Figure 3:**
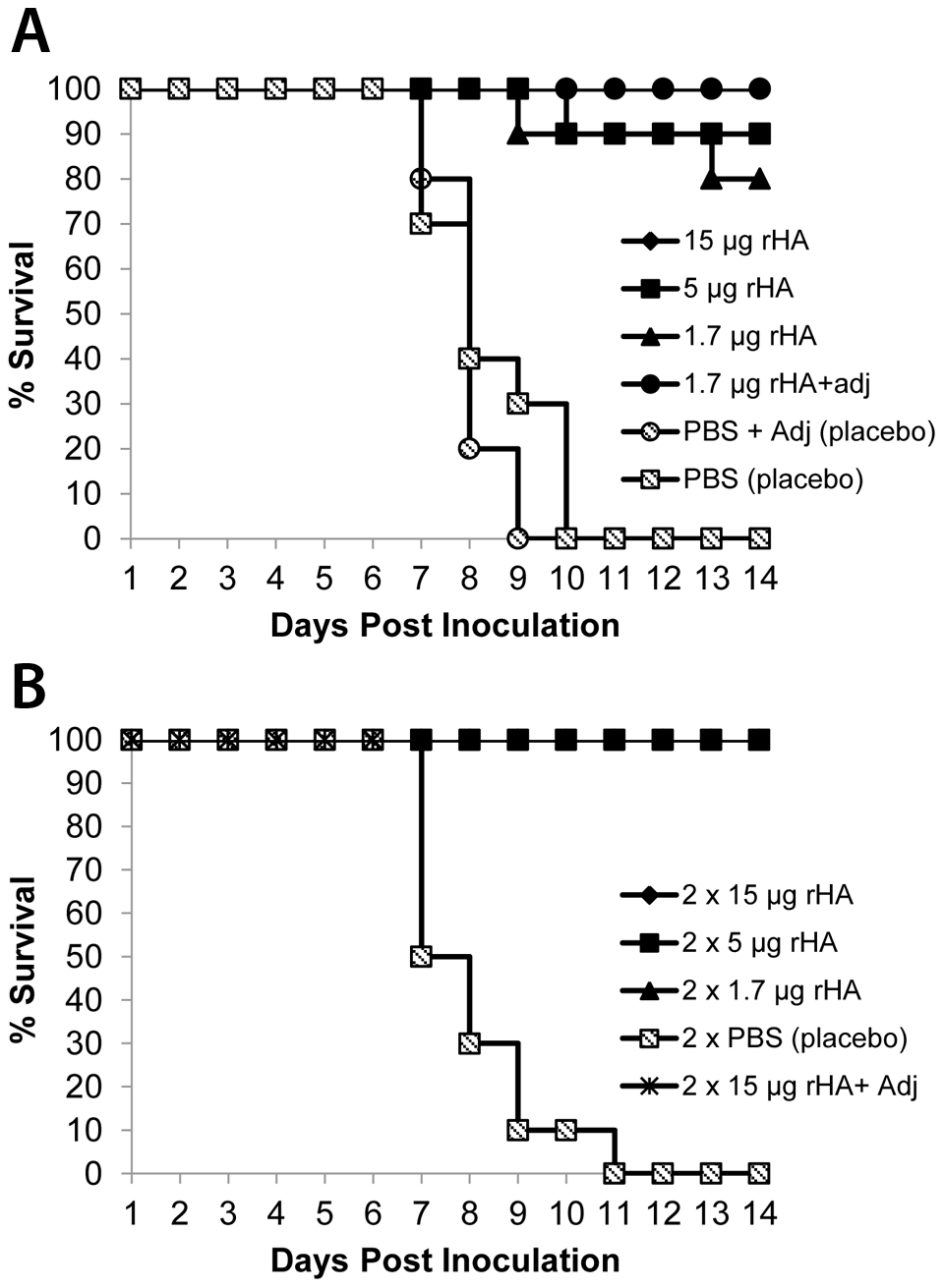
Effect of vaccination dosage and regimen on survival after inoculation. Mice were given a single vaccination (A) or two vaccinations 21 days apart (B), challenged and their survival monitored for 14 days. N = 10 mice per group.

**Figure 4:**
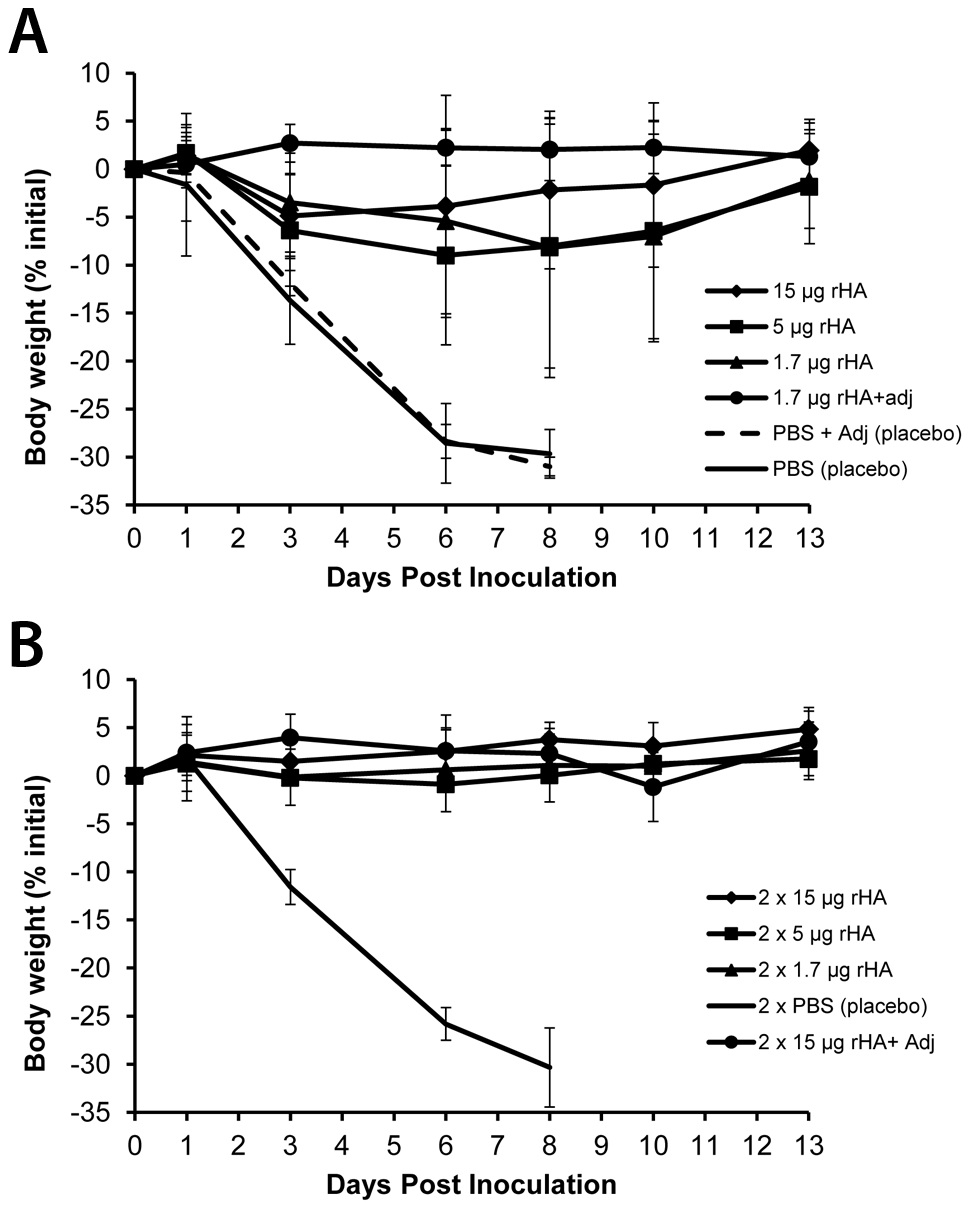
Effect of vaccination dosage and regimen on body weight after inoculation. Mice were given a single vaccination (A) or two doses 21 days apart (B), challenged and their body weight monitored for 14 days. N = 10 mice per group.

Mice were separated into 11 groups according to the schedule presented in Table 2. Three groups were given single-dose immunizations without adjuvant, and one group was given a single dose immunization with adjuvant. Similarly, three groups were given two doses (3 weeks apart) and one was given two doses (3 weeks apart) with adjuvant. Adjuvant was used with two different groups according to differing rationales. Because this study represents the first parenteral use of a *Schizochytrium*-derived preparation, adjuvant use with the highest, two-dose group was intended to provide an upper range of measurable responses (group 11). Adjuvant effects could be more clearly tested by establishment of parallel groups receiving the lowest rHA dosage with and without adjuvant (groups 4 and 3 respectively). All remaining groups served as placebo controls. Mice were monitored for survival and weighed for 14 days after lethal challenge with PR8 (Figures 3 and 4 respectively).

**Table 2:** Mouse challenge study design.

Group	rHA (µg)	Adj	Vaccination (day); n = 16	Challenge (day); n = 16	Blood*(day); n = 16	BAL**(day); n = 1–3
1	15	−	21	42	21, 42, 56	45, 49
2	5	−	21	42	21, 42, 56	45, 49
3	1.7	−	21	42	21, 42, 56	45, 49
4	1.7	**+**	21	42	21, 42, 56	45, 49
5	PBS	**+**	21	42	21, 42, 56	45, 49
6	PBS	−	21	42	21, 42, 56	45, 49
7	15	−	0, 21	42	0, 21, 42, 56	45, 49
8	5	−	0, 21	42	0, 21, 42, 56	45, 49
9	1.7	−	0, 21	42	0, 21, 42, 56	45, 49
10	PBS	−	0, 21	42	0, 21, 42, 56	45, 49
11	15	**+**	0, 21	42	0, 21, 42, 56	45, 49

On the indicated days, 11 groups of mice (n = 16 per group) were vaccinated with rHA and challenged with infectious PR8 virus.

* All mice were bled for sera on the indicated days and all survivors were bled at study conclusion (Blood).

** Some mice (n = 1–3) were sacrificed for bronchoalveolar lavage (BAL). Phosphate buffer saline (PBS), AddaVax™ adjuvant (Adj).

Among the groups receiving single dose vaccinations without adjuvant, protection correlated with dose level; 80% survival with 1.7 µg rHA, 90% with 5 µg rHA, and 100% with 15 µg rHA (groups 3, 2 and 1, respectively). All surviving mice from these groups initially lost some weight (as low as ∼23% of their initial body mass) but eventually recovered, with the exception of three animals; one from group 2 and one from group 3 died on day 9, with the last death in group 3 on day 13. Mice in groups receiving 2 doses of rHA without adjuvant all survived regardless of dose level (1.7, 5 or 15 µg). All mice vaccinated with rHA+adjuvant (groups 4 and 11) also survived challenge. As expected, mice receiving any of the placebo formulations died between days 7 and 11 (groups 5, 6 and 10). Mice given 1.7 µg of vaccine with adjuvant (group 4) realized slight gains in average weight during the 14 days post-challenge. Similarly, all mice from the groups receiving two doses of vaccine either gained or retained weight after virus challenge (groups 7–9 and 11).

### Virus Titers

**Figure 5:**
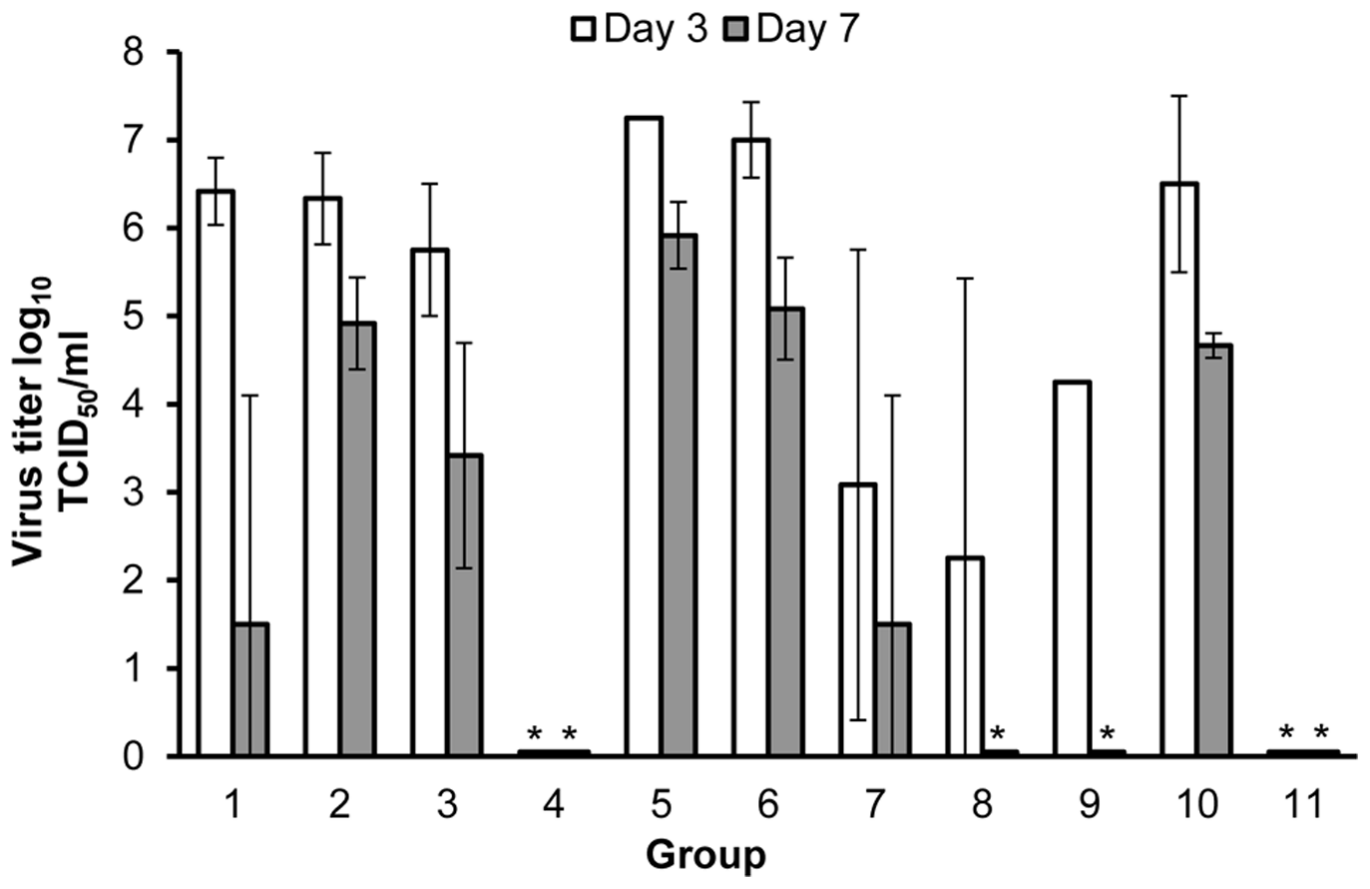
Virus titers in the lung of vaccinated and control mice after challenge with A/Puerto Rico/8/1934 (H1N1) virus. Mice were euthanized on day 3 (white bars) and day 7 (gray bars) and brochoalveolar lavage (BAL) washes were collected. Titers are given as the averages of three BAL washes except for those harvested from group 8 on day 3 and 7 and from groups 5 and 9 on day 3, for which only 2 BAL were available. Only one BAL was harvested from group 9 on day 3. Groups 1–6 were given single vaccinations. Groups 1–8 were given two vaccinations (three weeks apart) of the same mass with each injection. Groups 1, 7, and 11 were given 15 µg rHA, groups 2 and 8 were given 5 µg rHA, and groups 3, 4, and 9 were given 1.7 µg HA. Groups 5, 6, and 10 were given placebo injections. Groups 4, 5, and 11 were given vaccinations formulated with adjuvant. Results are shown as mean ± SD virus titers in (BAL) washes. *Statistically significant decreases in titers as compared with placebo are indicated. Limit of detection is 10^1.5^ TCID_50_/ml.

Infectious virus titers (log_10_ TCID_50_/ml) from samples of bronchoalveolar lavage (BAL) recovered on days 3 and 7 post-challenge are shown in Figure 5. Titers from day 3 were measureable in all groups. Groups vaccinated with rHA+adjuvant (4 and 11) had virus titers several logs lower than groups vaccinated with rHA alone or placebo. Small sample sizes (group 9, n = 1) precluded a statistical evaluation for day 3 BAL samples across the study groups. For all groups, virus titers appeared lower at day 7 post-challenge than at day 3 post-challenge. By day 7, virus had been completely cleared in the groups receiving two doses of 5 and 1.7 µg of HA, but not in the 15 µg group (significant variation among individual titer values). Virus could not be detected in cohorts receiving either of the adjuvant-formulated vaccines (groups 4 and 11) at days 3 and 7 post-challenge, indicating that the mice in these groups were unable to establish a persistent, replicating viral infection in the lungs. Two vaccinations (with or without adjuvant) or a single vaccination (with adjuvant) significantly decreased viral titers by day 7 when compared to controls (p<0.05; ANOVA).

### Serological Response: Hemagglutination Inhibition

To evaluate the presence of HA-specific antibodies, the hemagglutination inhibition (HI) activity of collected sera was determined (Table 3). The highest HI titers were found in the groups receiving adjuvant-formulated vaccine (groups 4 and 11). HI titers increased significantly after the second injection (for applicable groups). HI titers increased in all groups after infectious challenge, with the exception of group 11, for which HI titers dropped moderately after challenge (HI GMT 1225 to 787 from day 42 to 56). For those groups receiving 2 vaccinations, day 42 HI sero-response rates were between 25 and 100%. Among the single-dose groups at day 42, the majority of mice (49/52) had no detectable HI titer (≥40), with the animals receiving 1.7 µg of rHA+adjuvant having the highest sero-response rate (12.5% 2/16). Interestingly, in the absence of detectable HI titers, mice were protected from lethality.

**Table 3:** Hemagglutination inhibition activity against homologous virus post-vaccination and post-challenge.

Group	rHA (µg)	Adj	Day 0 HI	Day 21 HI	Day 42* HI	Day 56 HI
**1**	15	−	−	<10	<10	10/10 (40–640; 218)
**2**	5	−	−	<10	<10	9/9 (160–640; 320)
**3**	1.7	−	−	<10	1/16 (10; 10)	8/8 (80–640; 269)
**4**	1.7	**+**	−	<10	2/16 (80–160; 113)	10/10 (320–1280; 640)
**5**	PBS	**+**	−	<10	<10	n. t.
**6**	PBS	−	−	<10	<10	n. t.
**7**	15	−	<10	2/16 (20–40; 28)	12/16 (20–320; 75)#	10/10 (160–640; 298)
**8**	5	−	<10	<10	4/16 (20–160; 48)#	10/10 (80–320; 196)
**9**	1.7	−	<10	2/15 (10–20; 14)	13/16 (40–320; 61)#	10/10 (160–640; 393)
**10**	PBS	−	<10	<10	<10	n. t.
**11**	15	**+**	<10	16/16 (40–640; 153) #	14/14 (640–5120; 1225) #	10/10 (640–1280; 787)

HI antibody titers were determined against A/Puerto Rico/8/1934 (H1N1) virus and are expressed as the reciprocal of the highest dilution of sera which inhibited hemagglutination of 4 HA units of virus. All values are expressed as geometric mean titers (GMT) of inhibiting sera from dilutions which offered a response. The numbers of positive sera are indicated as the number of positive sera/total number of sera. The range of titers and the GMT of the positive sera are listed in parentheses. HI titers less than 10 were below the limits of detection. Serum samples were not taken from mice which did not survive challenge (n. t.) or from mice which had not yet been vaccinated with either placebo or experimental formulations (–).

# HI titers were significantly higher when compared to prime only doses.

* Day 42 = challenge.

## Discussion

A novel system for the production of transgenic proteins was used to express several hemagglutinin genes of influenza virus. This investigation describes the first use of the *Schizochytrium* platform for the expression of recombinant proteins for commercial pharmaceutical or veterinary applications. The four HA proteins reported in this study are derived from strains of Influenza A and B, and Influenza A hemagglutinin subtypes H1 and H5. The hemagglutination assay was used for simple and rapid detection of HA expression for all initial screens. Factors associated with variation in HA activity levels included HA subtype (H1 vs. H5), individual HA transformants, and culture conditions (fermentors vs. flasks). We chose to characterize one HA derived from influenza PR8 in greater depth due to the availability of an acceptable animal model for challenge with homologous virus and because of the broad use of this isolate in both laboratory and industrial contexts.

The expression of full-length HA as an extracellular protein without the aid of the matrix protein, viral vectors, or any agent which might facilitate the budding of virus-like particles for which other platforms are well-known, is a notable result [4]. In some systems, for efficient, extracellular expression of HA, the C-terminal membrane spanning domain is necessarily truncated [6], [34]–[37]. The ability of *Schizochytrium* to facilitate export of membrane-bound HA however, obviates the need for truncated forms. One potential mechanism for this may involve filamentous extensions from the main cell body of many other Thraustochytriales under certain conditions. These filopodia-like structures are produced by unique organelles called bothrosomes which assemble actin polymers and extrude them through the cell wall, while retaining a coating of outer membrane lipids and embedded membrane proteins [38]. The role these structures play in HA export is a subject for continuing investigation.

Although cleavage of HA0 to HA1 and HA2 subunits is not required to elicit immunity or hemagglutination, it is required for membrane fusion and influenza infectivity [39]. Therefore cleavage of HA may yield an antigen offering more conformational epitopes for the generation of functional neutralizing antibodies. HA cleavage, presumably by an endogenous *Schizochytrium* protease, was characterized by the introduction of a point mutation in a region of the PR8 HA gene encoding the cleavage recognition site. This mutation prevented HA cleavage and verified that the event occurs at exactly the same site as in native influenza hosts, suggesting that the antigenic conformation of HA from *Schizochytrium* might closely mimic that of HA produced by natural hosts. This feature is apparently not shared by certain other systems used to express transgenic influenza HA, such as fungi and higher plants.

Native PR8 HA possesses 6 glycosylation sites and like all influenza strains, glycoforms found on envelope proteins are generally devoid of terminal sialic acid residues by the action of neuraminidase. It is thought that the desialylation of host glycoproteins prevents virus aggregation and re-infection of an infected host cell, thereby improving the efficiency of the infection cycle [40]. Although the NA of PR8 has been successfully expressed using *Schizochytrium* (both independently and as a co-expression product with HA, data not shown) its presence was not required for expression of HA, since *Schizochytrium* does not synthesize or attach terminal sialyl residues to protein glycoforms which could induce HA aggregation. We detected only trace amounts of hypermannose glycans of the type found on yeast proteins, and a majority of high-mannose glycoforms. These latter glycoforms may be advantageous for vaccine design as other studies have demonstrated that similar glycan structures expose the HA molecule to neutralizing antibodies and receptor ligands to a greater degree, and that HA-based vaccines become more protective with correspondingly simpler HA glycostructures [13], [41]. Similar high-mannose glycans are also found among glycoproteins expressed by lepidopteran expression systems, with the exception that these glycoforms usually also contain a core fucose residue [42], [43]. Our analyses did not reveal any fucose residues; or xylose residues commonly associated with plant and green algal glycans [44]. However, the high-mannose structures which were observed, such as (GlcNAc)_2_(Man)_5–6_, are assembled in part by α1,3 and α1,6 linkages which have been demonstrated to be immunogenic in mammals [45]. The glycan (GlcNAc)_2_(Man)_6_ is also found at Asn 65 of the HA1 subunit from mammalian cells infected with influenza [13] although it is unclear to what degree these α1,3 mannoses may promote immunity to viral infection when associated with vaccine antigens.

To determine whether PR8 HA produced by *Schizochytrium* could serve as an effective vaccine, the BALB/C murine challenge model was used. The results show that HA, as expressed from *Schizochytrium*, enabled mice to mount an immune response which protected against subsequent lethal challenge with live homologous influenza virus. Health and survival of groups given two rHA vaccinations (100%) or rHA with a squalene adjuvant (100%) was unequivocal. Robust HI titers were observed in all vaccinated mice by day 56 (14 days post-challenge) although HI titers on day 42 were not clearly correlated with protection, justifying additional investigation of immune mechanisms in this model. All mice receiving two doses or adjuvant-formulated vaccine gained weight over the course of the study, including cohorts receiving the lowest dose of HA. No mice among any placebo group survived viral challenge, while 80% of mice given a single injection of the lowest dose of HA without adjuvant survived the infection. Taken together the results from this murine model indicate that recombinant influenza HA, as expressed by *Schizochytrium*, functions as an effective vaccine against lethal influenza virus challenge, comparing favorably with proof of concept studies reported for other transgenic expression systems [5], [36], [46]. Further studies will be required to evaluate the feasibility of *Schizochytrium*-produced influenza vaccine candidates purified and formulated for advanced non-clinical and eventual clinical development.

Cell-based systems, paired with synthetic biology, may be beneficial alternatives to egg-based manufacturing. Synthetic biology provides greater flexibility for transgenic systems via rigorous codon optimization techniques and RNA structure prediction; allowing for not only higher levels of protein expression but greater probability of expression for a broader range of targets. The scalability and yield of systems for expression of influenza proteins are also important to consider. Although *Schizochytrium* is routinely grown in 250,000 l fermentors, the existing process is designed for production of lipids. On a mass basis, HA yields from *Schizochytrium* shake flask cultures of 5–20 mg/l have been observed. These levels are comparable or better than reported HA yields from other eukaryotic recombinant systems [36]. The need for purification of unrelated viral material (such as baculovirus) is also obviated by the *Schizochytrium* platform. Similar to the baculovirus system, *Schizochytrium* is capable of producing an HA molecule with an intact transmembrane domain which is free of fusion partners or truncations that may reduce immune specificity but without the risks of virus-vectored expression. These traits justify continued development and evaluation of the *Schizochytrium* platform as an alternative to conventional systems not only for influenza antigens but other recombinant proteins for vaccine and therapeutic applications.
